# Children’s agency in the family, in school and in society: implications for health and well-being

**DOI:** 10.1080/17482631.2019.1634414

**Published:** 2019-10-14

**Authors:** Emma Sorbring, Leon Kuczynski

**Affiliations:** University West, Sweden; University of Guelph, Canada

As guest editors, we would like to introduce the eight articles included in the thematic cluster Children’s agency in the family, in school and in society: implications for health and well-being, in the *International Journal of Qualitative Studies on Health and Well-being*. The idea that children are active and influential agents in family life, has slowly gained acceptance since the 1970’s when researchers recognized that the causal effects of children on parents need to be taken into account in processes such as socialization (Bell, 1968) and human development (Sameroff, 1975). Since then, the nature, and scope of child agency has been elaborated in both sociology (James, Jenks, & Prout, 1998) and developmental psychology (Kuczynski, 2003; Kuczynski & De Mol, 2015). Currently, the idea that children are actors and agents who contribute positively to family and other social processes is well accepted at a theoretical level. However, the idea of child agency has been slower to take root in social policy, family interventions, and social services. In these applied arenas, the implications of children’s influence and capacity as agents has been neglected, discounted, or viewed from a problem-focused lens. The articles in the thematic cluster address this gap by illustrating the growing understanding that children contribute constructively to the diverse processes in which they are involved and arguing that researchers, clinicians, teachers, and social workers must engage or accommodate children’s perspectives and initiatives in order to promote children’s health and well-being. In this introduction we discuss the articles with respect to the breadth of contexts for child agency that they sample, the advances in the underlying concepts of child agency that they represent, and their implications for health and well-being.

## Contexts of children’s agency

Together, the articles in this thematic cluster describe children’s capacities to act constructively on their own behalf and on behalf of others in a broad range of family, school and societal contexts. Four of the articles focus on children’s agency in relation to care and health-care organizations, using clinical and non-clinical samples. For example, Bolin (2019) considers children as interpretive agents with legitimate knowledge whose participation should be engaged by service providers when making of decisions in programs of care. Wickström (2019) documents adolescent girls’ evasion, resistance and repurposing of psycho-educational program exercises to suit their own needs and preferences. De Mol, d’Alcantara, and Cresti (2019) examine the capacity of clinically depressed adolescents to identify cultural expectations that detrimentally influence their views of themselves and their possibilities for success. Robson and Kuczynski (2019) using a clinical sample report that mothers of hard to manage, aggressive children make qualitative distinctions between different forms of noncompliance based on their perceptions of whether children fully act as intentional agents when resisting their requests.

The remaining four articles, have a greater focus on family and interpersonal processes, but in relation to applied arenas such as social policy, institutions and services. Kuczynski, Pitman, and Twigger (2019) present a fine grain analysis of school age children’s strategies for expressing resistance to unwanted parental requests that suggest a multifaceted approach for clinically assessing an agentic behavior that has traditionally been viewed as problematic and in need of suppression. Bergnehr (2019), with refugee families, and Cheang and Goh (2019) with low-income families, explore the capacity of children to act on behalf of themselves and to contribute positively to their families’ well-being. Finally, Gurdal and Sorbring (2019) examine children’s perception of their own agency in different relationship contexts, including the family, at school, and with peers.

## Perspectives on children’s agency

The present articles demonstrate advances in the conceptualization of children’s agency that highlights the importance of context for children’s experience and manifestation of their agency. Historically two different approaches, one relatively individualistic and one relatively social, presented contrasting perspectives on children’s agency that continue in some form today (Kuczynski, Harach, & Bernardini, 1999; Morrow, 2003).

Individualistic perspectives are evident within developmental theories such as Piaget’s theory of cognitive development that assume that children are living organisms with inherent capacities to self-regulate, self-organize, and actively engage with their environments. As captured by the ontological metaphor of *becoming*, this is an optimistic “can do” perspective on children’s capacities as agents in the transformative process of development (Overton, 2015). In contrast, social approaches to child agency evident in sociological and feminist research on family life (Morrow, 2003) had a more ambivalent perspective on human agency in general and on child agency in particular. Framed, as the tension between agency and structure, some social perspectives take the form of acknowledging human agency, but focusing on the ways social structures constrain and channel how individuals can deploy their actions. Social approaches specialize in analyzing with great complexity how structural contexts including culture, society, institutions, and power inequalities limit the possibilities of individual agents to create something new or to act effectively to influence or change their social contexts. In the present issue, the contributors used more contemporary theoretical frameworks of *social relational theory* (Kuczynski, 2003; Kuczynski & De Mol, 2015) and the *sociology of childhood* (James et al., 1998; Morrow, 2003) that better integrate individualistic and social approaches.

*Social relational theory* (Kuczynski & De Mol, 2015) approaches the construct of children’s agency from the perspective of family dynamics and interpersonal processes in social relationships. Social relational theory originated in attempts to understand accumulating empirical findings on children’s effects on parents as well as everyday family phenomena such as children’s noncompliance to parental demands, parent-child conflict, and parents’ receptivity to children’s influence. Although pervasive and taken for granted, these social dynamics were not comprehensible under the traditional unilateral assumptions regarding the nature of agency, power, and direction of influence in the family (Kuczynski, 2003). According to social relational theory, parents and children are equally agents, influence between parents and children is bidirectional and nonlinear, and the dynamics of human agency, interpersonal influence, and asymmetrical power needs to be understood in the context of the interdependent, long-term parent-child relationship. In addition to providing a comprehensive analysis of children’s capacities as agents, social relational theory seeks to understand how close relationship contexts enable children’s agency, not only constrain it.

*The sociology of childhood* (James et al., 1998; Morrow, 2003) addressed the traditional neglect of children and children’s agency within sociology but maintained a strong contextual perspective for understanding children’s agency. Using the ontological metaphor of *being*, the sociology of childhood focuses on children’s present, situated experience not as children in the process of developing. Children are viewed as actors whose experiences are studied with a purpose that is independent of the perspectives and agendas of adults. Methodologically, important foci of this approach include studying the experiences of children from their own perspective, documenting diversity of childhood experiences, and analyzing the socially constructed meanings that impact on children’s experiences (Morrow, 2003).

Consistent with these new theoretical frameworks, what stands out in this collection of articles is a consensus on the importance of context in understanding children’s agency. First, there is a diversity of contexts where children act as agents. These include the macro level contexts of culture (De Mol et al., 2019) and acculturation (Bergnehr, 2019), poverty (Cheang and Goh (2019), school (Bolin, 2019; Wickström, 2019) and dyadic family relationships Kuczynski et al. (2019), Robson and Kuczynski (2019), Gurdal and Sorbring (2019). Nowhere to be found is the individual agent operating devoid of context.

We can also notice an increasing complexity in understanding the dynamics underlying of intersections of context and agency. For example, De Mol et al. (2019) unpack the multi-layered messages that adolescents derive from the social representations of depression in society. Wickström (2019) provides a multifaceted analysis of the context of psychoeducational school programs using Deleuze and Guattari (1987) theories of assemblages. In her analysis, social contexts have multiple material aspects, social meanings and expectations that dynamically change and alter the possibilities for action, including resistance and cooperation by human agents.

Particularly well represented was a consideration of relationship contexts of agency the idea that children’s experience and practice of agency is relational not individual in nature. In other words, how children experience themselves as agents, how they express themselves as agents and the extent to which their actions as agents are constrained or enabled depends on the specific interpersonal relationships in which they interact with other social agents. This is most clearly illustrated by Gurdal and Sorbring (2019) who find that children’s sense of efficacy, or belief that they can influence another person and their choice of social influence strategies depended on whether they acted within parent-child relationships, peer-relationships or institutional teacher-pupil relationships. Kuczynski et al. (2019) and Robson and Kuczynski (2019) consider unique features of the parent-child relationship that affords children leeway to express their resistance despite their difference in power in an asymmetrical but interdependent close relationship. Cheang and Goh (2019) attribute the ability of children to succeed academically despite living in disadvantaged, low income families, to the fact that they are connected agents rather than isolated agents. Being a connected agent means that children have social resources that they can draw on to empower their effectiveness as agents. It also means that their connection to others creates relational goals that propel children to act not just in their own interest as individuals but also to act in a way that benefits their parents and the family as a whole. Thus, relationship contexts support and enable children’s agency and give it direction.

## Policy and practice

The gap between academic theorizing and empirical research, on the one hand, and application by designers of social policy and service providers, on the other, is well known. To address this gap each of the contributors described the implications of their research on children’s agency for health and well-being. The significance of the present contributions is best viewed against a backdrop of traditional views that children are the passive objects of social services. Thus, it is common for service providers to perceive children’s agentic manifestations as problems that must be fixed or obstacles that must be handled or circumvented, and whose potential to contribute constructively to social interventions and therapeutic programs are unrecognized or ignored.

Instead, the present articles highlight the possible contributions of children and young people to the services that they receive. Bolin (2019) argues that acknowledging children’s agency when organizing child care services contributes to everyday practice routines that helps children and their families in a more effective way. Children’s agency in family interventions should be harnessed so children can contribute to the remedial process and not just resist it. The results from De Mol et al. (2019) and Robson and Kuczynski (2019) are useful for work with clinical populations. Although working with children and young people with challenging behavior or depression means focusing on intrapersonal processes, these articles illustrate that children receiving services are agents with their own legitimate perspectives not objects on whom services can be imposed. As well, these articles foster a strengths perspective that reinterprets children’s behaviors in a constructive way that previously were interpreted as deviant. Wickström (2019) questions the predominant framing of psychological health programs, highlighting young people’s own views about psychological health. Furthermore, a common, but misleading view, is that young people living in precarious situations, are passive and helpless victims. Instead, recognizing that even children experiencing adversity or oppression are agents who are capable of acting in a way contributes to their own well-being and the well-being of their families. The results from Bergnehr (2019), in the case of refugee families, Cheang and Goh (2019) in the case of economically disadvantaged families, and Wickström (2019) in the case of children with mental health concerns, will help social services professionals to develop skills to identify children’s acts of agency despite disadvantage and to work with or support children’s capacities and initiatives to achieve therapeutic goals. Furthermore, incorporating children’s and young people’s perceptions and knowledge into therapeutic interventions, is a way for professionals working with children and young to find proper ways to help young people on their own terms.

## Conclusion

We hope that this thematic cluster will supply the reader with a new and widened understanding of child agency and deepen their insight into the importance of child agency in health and well-being. The articles identify diverse contexts inside the family and in society where children deploy their strategic and interpretive capacities to achieve their self-chosen goals. The articles illustrate how new theoretical frameworks emphasize the contextual and relational nature of agentic processes. This convergence of interest in both social and individual processes for understanding children’s agency is welcome and invites interdisciplinary collaboration in research on the practical implications of children’s agency. Finally, the articles provide directions to service providers and designers of social policy for considering children’s agentic capacities and perspectives as a resource for their own work with children and their families. The larger implication is that children’s agency can be supported with the goal of empowering children to feel and act as resourceful and skillful agents so they have a positive impact on their own health, well-being.
